# Integration of Family Planning Counselling to Mass Screening Campaign for Cervical Cancer: Experience from Guinea

**DOI:** 10.1155/2018/3712948

**Published:** 2018-03-11

**Authors:** D. W. A. Leno, F. D. Diallo, A. Delamou, F. D. Komano, M. Magassouba, D. Niamy, J. Tolno, N. Keita

**Affiliations:** ^1^Regional Francophone Center for Training in the Prevention of Gynecological Cancers (CERFFO-PCG), Conakry, Guinea; ^2^Public Health Department, Gamal Abdel Nasser University of Conakry, Conakry, Guinea

## Abstract

**Aim:**

To assess feasibility of integrating family planning counselling into mass screening for cervical cancer in Guinea.

**Methodology:**

This was a descriptive cross-sectional study conducted over a month in Guinea regional capital cities. The targeted population comprised women aged 15 to 49 years. Nearly 4000 women were expected for the screening campaigns that utilized VIA and VIL methods with confirmation of positive tests through biopsy. A local treatment was immediately performed when the patient was eligible.

**Results:**

Overall 5673 women aged 15 to 60 years were received, a surplus of 42% of the expected population. 92.3% of women were aged 15–49 years and 90.1% were 25–49 years. Long-acting methods were the most utilized (89.2% of family planning users). 154 precancerous and cancerous lesions were screened, a global positivity rate of 2.7%.

**Conclusion:**

Integration of counselling and family planning services provision during cervical cancer mass screening is a feasible strategy. A cost-effective analysis of this approach would help a better planning of future campaigns and its replication in other contexts.

## 1. Introduction

Family planning (FP) and cervical cancer screening are two strategic areas of the fighting against maternal and infant morbidity and mortality and the death of women in resource-poor countries [1, 2].

Nearly half of the world's maternal deaths occur in sub-Saharan Africa, where one of 31 women is at risk of dying from pregnancy and childbirth complications [3, 4]. Two-thirds of these deaths could be avoided if FP needs were met [1]. FP is known as one of the most important and cheapest interventions to reduce maternal morbidity and mortality rates [5]. However, its access and use are still limited in several developing countries, especially in rural areas [6].

Cervical cancer generally occurs in developing countries (80%) and is the leading cause of cancer deaths among women in Africa. It usually occurs at the age of full sexual activity [7, 8]. The rate of uterine cancer remains high in most developing countries because of limited access to health services, ignorance, and the lack of cancer screening and treatment programs [2]. The World Health Organization (WHO) estimated in 2009 that, approximately, 5% of women was screened for cervical cancer in resource-poor settings, compared to 40–50% in developed countries [9]. However, cervical cancer can be prevented by screening and treatment of precancerous lesions [10].

Guinea has one of the lowest indicators of maternal and child health in Africa, with 740 maternal deaths per 100,000 live births and 91 infant deaths per 1000 live births in 2012 [11]. The fertility rate was estimated at 5.7 children per woman in the same year, with a contraceptive prevalence of 9% with 6% for modern contraceptive methods in the same year.

In addition, cervical cancer, accounting for 50% of cancers in Guinea, is one of the main health problems of women. It is the first gynecological cancer of Guinean woman, with an incidence of 56.3 per 100,000 [4]. The Guinean context of family planning and prevention and control of cervical cancer is not different from that of other developing countries. Improving these health indicators in the country requires appropriate strategies to improve women's access to family planning and prevention and treatment of cervical cancer. The present study consisted of experiencing an approach to improve women's access to these services. These included planning services integrated with a community-based cervical cancer screening campaign in the administrative regions of the country.

The study aimed at assessing the feasibility of integrating family planning services (counseling and contraceptive care) to mass screening for cervical cancer in Guinea.

## 2. Methodology

### 2.1. Type and Study Period

This was a descriptive cross-sectional study of one month (21st October to 22nd November 2013).

### 2.2. Camp Sites

This campaign covered the eight [8] administrative region capitals of Guinea: Conakry, Boké, Kindia, Mamou, Labé, Faranah, Kankan, and Nzérékoré. FP services and cervical cancer screening sessions took place at the CERFFO-PCG (French Regional Training Center for the Prevention of Gynecological Cancers) located in the Donka National Hospital in Conakry and the seven regional maternal centers in the cities mentioned above.

### 2.3. Study Population

The study population consisted of women aged between 15 and 49 years for family planning (FP) and women aged between 25 and 49 years for cervical cancer screening. All women who were not pregnant and had not undergone a total hysterectomy and who gave informed consent for participation in the study were included.

### 2.4. Study Procedures

Cervical cancer screening and promotion of contraceptive methods were preceded by information and awareness campaigns carried out through national and local media, posters and banners, and through health facilities. After the registration of women admitted to the screening services, a counseling group in French and national languages on the relevance of FP and cervical cancer screening was carried out. Women who accepted the principle of cervical cancer screening participated in a new individual counseling session prior to obtaining informed consent. An anonymous questionnaire was then administered to women to collect sociodemographic information on their reproductive health and sexual life. Pending her turn for the cervical cancer screening test, the woman was referred to one of the PF's interpersonal counseling offices. If a FP method was accepted, it was immediately dispensed to the woman. On a deliberate basis, the promotion of long-lasting contraceptive methods was strengthened during this study.

### 2.5. Cancer Screening

The method of visual inspection of the cervix after 5% acetic acid (VIA) application and visual inspection of the cervix after application of lugol (VIL) was used to screen for cervical cancer. Screening was performed during the gynecological examination. It began by inspecting the vulvar region in search of lesion. After placing a speculum, the squamocolumnar junction zone (SJZ) was previously identified. An unprepared test was initially performed, followed by VIA and VIL then by a biopsy if the test was positive. The VIA consisted of applying a 5% solution of acetic acid to the cervix and under high light in observation after one minute of color changes at the SJZ level. Negative VIA patients show no change in color. On the other hand, positive VIA patients present a dense, acidophilic, well-defined whitish color near or within the SJZ [12–14]. In VIL, it consisted of the application of iodized solution on the cervix and then observing the color changes of the SJZ. In negative VIL patients, coloration of black or mahogany colored was observed. In the case of positive VIL, the SJZ is colored in saffron or mustard yellow [12–14]. In the event of a positivity of one of the tests, a colposcopy was performed followed by a biopsy at the positive zone. The biopsy site was marked on the chart of the questionnaire, and the specimen was collected in a small vial containing formalin and labeled with the woman's identification number and date of collection before being sent to the pathology-anatomy laboratory of the Donka National Hospital. Subsequently, the patients were immediately treated either by cryotherapy or by cold coagulation, with the exception of women with large lesions not eligible for immediate treatment who were reconvened for appropriate treatment according to the country's protocols. All women seen in colposcopy were informed of the need to be reviewed one year later for follow-up. Those treated were informed of the need to be reviewed within six weeks, three months, six months, and one year. For women with suspected cancer, an additional consultation was carried out to determine the stage of development, to carry out an assessment of extension, and to propose a scheme of management (surgery, radiotherapy, or palliative treatment).

### 2.6. Family Planning Offer

The provision of family planning methods was carried out in collaboration with the health workers of the campaign sites in order to facilitate the monitoring and the sustainability of the activities. After a group information session, women of reproductive age were received individually and then referred to a PF counseling room. They were given advice on the different methods of PF exposed on a “vanette.” The WHO disc for the choice of contraceptive methods according to medical admissibility criteria was also used. This was followed by a discussion between the woman and the health provider explaining the advantages and disadvantages of the different methods especially the method desired by the woman. During the counseling session, special emphasis was placed on the advantages of long-acting methods (LACMs). FP methods available during the campaign were essentially the copper intrauterine device (IUD), Jadelle implant, injectables, and pills (Microlut and Microgynon).

### 2.7. Data Analysis

A simple descriptive analysis was carried out with the SPSS version 17 software, and the results were expressed in percentages and mean values.

### 2.8. Ethical Considerations

The screening campaign project coupled with the family planning offer has been approved by the Ministry of Health and the National Ethics Committee for Health Research in Guinea.

## 3. Results

### 3.1. Preliminary Steps

A national campaign commission was settled up by the Ministry of Health. It was made up of three teams: a coordination team (led by the National Direction of Family Health and Nutrition of the Ministry of Health), a technical team (headed by the Regional Francophone Training Center for Prevention of Gynecological Cancers), and a communication and social mobilization team (led by the Health Promotion Division of the Ministry of Health). The capacities of the health providers composing the national technical team (18 doctors and midwives from the regions related to the campaign) were strengthened during a two-day training workshop in Conakry. The training focused on providing quality service for both campaign activities (FP and cervical cancer screening). The communication and social mobilization team carried out awareness-raising and community mobilization activities in the two weeks preceding the campaign at the national level (media) and at campaign site level (media and community activities at the level of health). Camp sites also received local training and were provided with reproductive health equipment, materials, and products to ensure efficient care and follow-up for seekers after the campaign.

### 3.2. Conduct of the Campaign

The campaign took place in two stages. The first consisted of a test campaign in the Boké region which mobilized the entire national technical team. This step identified the problems related to the implementation and coordination of activities and better planning the next steps. The second stage consisted of conducting the rest of the campaign in parallel in the other regions. The campaign lasted five days in Conakry and three days in the other regional capitals.

### 3.3. Campaign Population

A total of 5,673 women in the eight regions of the country participated in the cervical screening and family planning service delivery (Table 1). Participants were mostly adults of reproductive age (72.2%), followed by adolescents or young people (20.1%) and postmenopausal adults (7.7%). The distribution by age groups in each region was similar to that of the overall sample.

### 3.4. Prevalence of Cervical Cancer

**Figure 1:**
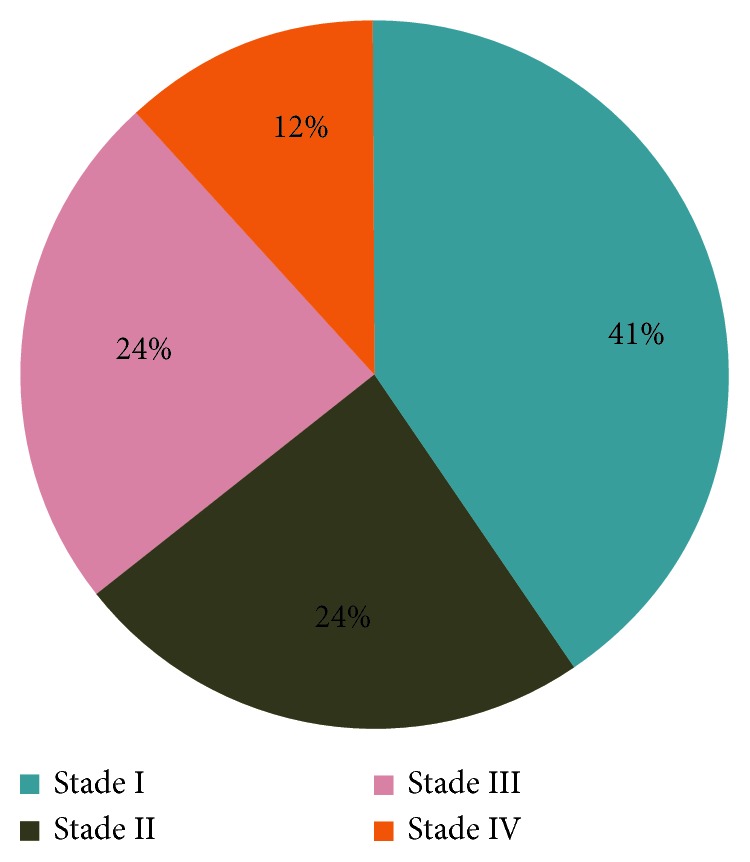
Distribution of cancer lesions according to the FIGO classification, in the cervical screening population, Guinea, October-November 2013 (*N* = 17); IUD: intrauterine device.

All campaign participants were screened for cervical cancer (Table 2). Precancer lesions were detected in 2.4% (*n*=137), with prevalence by age group of 1% for adolescents or young people (*n*=11), 2.8% for adults of reproductive age (*n*=113), and 3% in postmenopausal adults (*n*=13). The presence of cancer lesions was detected in 0.3% (*n*=17) of women, including one adolescent or young people (0.1%), 10 adults of reproductive age (0.2%), and 6 menopausal adults (1.4%). Among the cases of cancer detected, 7 (41%) were classified according to the FIGO classification in stage I, 4 (24%) in stage II, 4 (24%) in stage III, and 2 (12%) in stage IV (Figure 1).

### 3.5. Providing Family Planning Services and Contraceptive Prevalence

**Figure 2:**
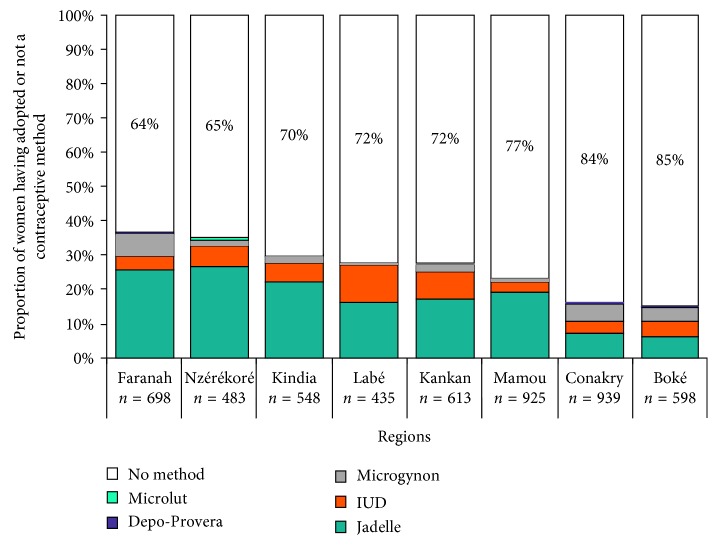
Contraceptive prevalence by region and contraceptive method in cervical screening population and family planning service delivery, Guinea, October-November 2013 (*N* = 5239).

FP service delivery was for women of reproductive age (*N* = 5239), with 92.3% of the campaign population (Table 3). All participants in this group attended an individual counseling session on modern contraceptive methods. As a result of counseling, 25.4% adopted a contraceptive method, of which 52.1% was adolescents or young people and 18% was adults of reproductive age. Long-lasting contraceptive methods were the most widely adopted methods: 86.9% for adolescents or youth and 85.9% for adults of reproductive age. Contraceptive prevalence varied by region and by contraceptive method (Figure 2). The Faranah region had the highest prevalence at 36%, followed by N'Zérékoré (35%), Kindia (30%), Labé and Kankan (28%), Mamou (23%), Conakry (16%), and Boké (15%). Implant, IUD, and Microgynon were the main contraceptive methods adopted.

## 4. Discussion

During this campaign, the expected results in terms of target women were exceeded. This shows that the strategy of integrating counseling and providing family planning services to mass screening for cervical cancer is a feasible strategy in the Guinean context. Our findings also show the existence of unmet needs for family planning and cervical cancer screening services at the local level. Cervical cancer screening is not systematic in the country's regional and prefectural health facilities due to inadequate equipment, training, and staff motivation, all which are necessary to ensure screening but also correct handling of detected cases.

As for family planning, the 2012 Demographic and Health Survey shows that 24% of women aged from 15 to 49 years have an unmet need for family planning [11], due in part to the inadequate public commitment in financing the sector. The study showed a difference between regions in terms of proportion of adolescents and young people in the sample. This difference may be explained by the fact that, in some regions, awareness-raising and mobilization sessions have targeted schools and universities that are full of teenagers and young people. Nevertheless, the good representation of this age group in the sample shows that adolescents and young people not only have high contraceptive needs but also an interest in using modern FP methods if they are offered to them.

Long-acting contraceptives (LACM) with the implant (Jadelle) and the intrauterine device (IUD) were the most prescribed during this campaign because of the importance they were given during the individual counseling sessions. Acceptance of LACM was high in all age groups and regions with a higher acceptance rate among adolescents and young people. This could be explained by the fact that this age group is at high risk of unwanted pregnancies on the one hand, but also because of the sociocultural context (stigmatization among others...), they prefer to use FP methods (IUDs, Jadelle) that do not require regular visits to health facilities.

During our campaign, 154 precancerous and cancerous lesions were found, representing an overall positivity rate of 2.7%. The fact that the majority of precancerous lesions have been found in women aged 25–49 may be explained by the fact that persistent infection with human papillomavirus (considered to be the main cause of cervical cancer) occurs often in young age. These findings that confirm the natural evolution of cervical cancer highlight the need to take the young population into account in cervical cancer screening programs in our work context, and on the other hand, need for secondary prevention through visual methods (VIA and/or VIL) and immediate treatment of precancerous lesions. This secondary prevention is all important as vaccination against the human papillomavirus remains very costly for our poor resource countries. On the other hand, the majority of cancer lesions were observed in women over 40 years of age (82.4% of the cancers found), which confirms the previous literature [15–17] that there is a lag of about ten years between the age of onset of invasive cancers and that of precancerous lesions. The screening of precancerous lesions and their immediate treatment is therefore a necessity to limit their evolution towards the stage of cancer. According to the literature, a woman undergoing screening for cervical cancer would reduce her risk of developing cervical cancer by 25–36% [17].

Our results show that if the majority of women with precancerous lesions have received immediate treatment, the management of cancer lesions is a challenge in Guinea because of its high cost and the time it takes to take it in charge. In Guinea, this care is taken in charge by the patient and her family without external help, which limits the choice of treatment. Finally, despite popular enthusiasm, this campaign was largely confined to women living in and around the regional capitals, which overall accounted for less than 1% of the women of reproductive age in the target regions. In this sense, the campaign excluded women living in rural areas, which raises questions about its equity.

In order to increase national coverage for cervical cancer screening and promote greater adoption of LACM, it is necessary to periodically conduct such campaigns in the country. To succeed, the collaboration of the staff of the implementation sites is essential and the definition of strategies of communication and mobilization of the women must precede the deployment of the technical teams. In the long term, it is necessary to envisage the integration of relocated campaigns at regional maternity level, preceded by the training of the personnel and the equipment of the structures.

## 5. Conclusion

This study shows that the coupled campaign of family planning and cervical cancer screening is feasible in the Guinean context. The acceptance of screening for cervical cancer and modern contraception, particularly LACM, was high. Our experience shows that creativity, good planning, and the organization of available resources can help reduce inequalities in access to FP and cervical cancer screening services in Guinea.

## Figures and Tables

**Table 1:** Age group and region of the women who participated in the screening campaign, Guinea, October-November 2013 (*N* = 5673).

Regions	Age groups			
	Teenagers or young people^∗^	Adults of reproductive age^∗∗^	Menopausal adults^∗∗∗^	Total
	*n* (%)	*n* (%)	*n* (%)	*n* (%)
Conakry	129 (12.1)	810 (76.2)	124 (11.7)	1063 (100)
Boké	128 (20)	470 (73.6)	41 (6.4)	639 (100)
Kindia	165 (28.7)	383 (66.6)	27 (4.7)	575 (100)
Mamou	170 (17.4)	755 (77.4)	51 (5.2)	976 (100)
Labé	67 (14.4)	368 (79.1)	30 (6.5)	465 (100)
Faranah	244 (33)	454 (61.4)	42 (5.7)	740 (100)
Kankan	105 (15.4)	508 (74.4)	70 (10.2)	683 (100)
Nzérékoré	134 (25.2)	349 (65.6)	49 (9.2)	532 (100)
Total	1142 (20.1)	4097 (72.2)	434 (7.7)	5673 (100)

^∗^15–24 years; ^∗∗^25–49 years; ^∗∗∗^50 years or more.

**Table 2:** Prevalence of cervical cancer by age group in cervical screening population, Guinea, October-November 2013 (*N* = 5673).

Cervical screening	Age groups			
	Teenagers or young people^∗^	Adults in reproductive age^∗∗^	Menopausal adults^∗∗∗^	Total
	*n* (%)	*n* (%)	*n* (%)	*n* (%)
Presence of precancerous lesions				
Yes	11 (1.0)	113 (2.8)	13 (3)	137 (2.4)
No	1131 (99.0)	3984 (97.2)	421 (97)	5536 (97.6)
Presence of cancerous lesions				
Yes	1 (0.1)	10 (0.2)	6 (1.4)	17 (0.3)
No	1141 (99.9)	4087 (99.8)	428 (98.6)	5656 (99.7)
Total	1142 (100)	4097 (100)	434 (100)	5673 (100)

^∗^15–24 years; ^∗∗^25–49 years; ^∗∗∗^50 years or more.

**Table 3:** Provision of family planning services and contraceptive prevalence by age group in the target population for family planning service delivery, Guinea, October-November 2013 (*N* = 5239).

Family planning services	Age groups		
	Teenagers or young people^∗^	Adults of reproductive age^∗∗^	Total
	*n* (%)	*n* (%)	*n* (%)
Counseling on modern contraceptive methods			
Yes	1142 (100)	4097 (100)	5239 (100)
Adoption of contraceptive method			
Yes	595 (52.1)	736 (18)	1331 (25.4)
No	547 (47.9)	3361 (82)	3908 (74.6)
Contraceptive methods adopted by duration of action (*N* = 1304)	*n*=595	*n*=736	*n*=1331
Long	517 (86.9)	632 (85.9)	1149 (86.3)
Short	78 (13.1)	104 (14.1)	182 (13.7)
Total	1142 (100)	4097 (100)	5239 (100)

^∗^15–24 years; ^∗∗^25–49 years.

## References

[1] WHO (2011). *Global and Regional Estimates of the Incidence of Unsafe Abortion and Associated Mortality in 2008*.

[2] WHO (2017). *Screening as Well as Vaccination is Essential in the Fight against Cervical Cancer*.

[3] World Health Organization (WHO) (2010). *Trends in Maternal Mortality: 1990 to 2008: Estimates Developed by WHO, UNICEF, UNFPA, and the World Bank*.

[4] WHO (2011). *Unsafe Abortion: Global and Regional Estimates of the Incidence of Unsafe Abortion and Associated Mortality in 2008*.

[5] Chola L., McGee S., Tugendhaft A., Buchmann E., Hofman K. (2015). Scaling up family planning to reduce maternal and child mortality: the potential costs and benefits of modern contraceptive use in South Africa. *PLoS One*.

[6] WHO (2016). *Family Planning/Contraception*.

[7] Arbyn M., Sankaranarayanan R., Muwonge R. (2008). Pooled analysis of the accuracy of five cervical cancer screening tests assessed in eleven studies in Africa and India. *International Journal of Cancer*.

[8] WHO (2009). Human papillomavirus vaccines: WHO position paper. *Weekly Epidemiological Record*.

[9] WHO (2009). Human papillomavirus vaccines: WHO position paper. *Biologicals*.

[10] Bayo S., Bosch F. X., de Sanjosé S. (2002). Risk factors of invasive cervical cancer in Mali. *International Journal of Epidemiology*.

[11] GUINEE (2013). *Enquête Démographique et de Santé et à Indicateurs Multiples (EDS-MICS 2012)*.

[12] Denny L., Quinn M., Sankaranarayanan R. (2006). Chapter 8: screening for cervical cancer in developing countries. *Vaccine*.

[13] Sankaranarayanan R., Wesley R., Thara S. (2003). Test characteristics of visual inspection with 4% acetic acid (VIA) and Lugol’s iodine (VILI) in cervical cancer screening in Kerala, India. *International Journal of Cancer*.

[14] Sellors J. W., Sankaranarayanan R. (2003). *Colposcopy and Treatment on Cervical Intraepithelial Neoplasia: A Beginners’ Manual*.

[15] ZurHausen H. (1996). Papillomavirus infections a major cause of human cancers. *Biochimica et Biophysica Acta*.

[16] Bosch F. X., Lorincz A., Munoz N., Meijer C. J., Shah K. V. (2002). The causal relation between human papillomavirus and cervical cancer. *Journal of Clinical Pathology*.

[17] Goldie S., Gaffikin L., Goldhaber-Fiebert J. (2005). Cost effectiveness of cervical screening in five developing countries. *New England Journal of Medicine*.

